# A study comparing outcomes between obese and nonobese patients with lumbar disc herniation undergoing surgery: a study of the Swedish National Quality Registry of 9979 patients

**DOI:** 10.1186/s12891-022-05884-8

**Published:** 2022-10-22

**Authors:** Niyaz Hareni, Fredrik Strömqvist, Björn E. Rosengren, Magnus K. Karlsson

**Affiliations:** 1 Departments Clinical Sciences and Orthopedics, Lund University, Skåne University Hospital, Inga Marie Nilssons gata 22, plan 4, 205 02 Malmö, Sweden; 2 Department of Orthopedics, Halland Hospital, Varberg, Sweden

**Keywords:** Lumbar disc herniation, BMI, Obesity

## Abstract

**Background:**

This study aimed to evaluate whether an increasing grade of obesity is associated with inferior outcomes after lumbar disc herniation (LDH) surgery.

**Methods:**

We retrieved data from the Swedish register for spine surgery regarding patients aged 20–64 who underwent LDH surgery from 2006–2016 and had preoperative and one-year postoperative data. A total of 4156 patients were normal weight, 4063 were overweight, 1384 had class I obesity, 317 had class II obesity and 59 had class III obesity (“morbid obesity”). Data included patient satisfaction, improvement in leg pain (assessed using the National Rating Scale; NRS; rating 0–10), disability (assessed using the Oswestry Disability Index; ODI; rating 0–100) and complications.

**Results:**

At one year postsurgery, 80% of normal-weight patients, 77% of overweight patients and 74% of obese patients (class I-III evaluated together) were satisfied (*p* < 0.001) [75%, 71%, 75% in obesity classes I, II, and III, respectively (*p* = 0.43)]. On average, all groups improved by more than the minimal clinically important difference (MCID) in both NRS leg pain (> 3.5) and ODI (> 20). NRS leg pain improved by 4.8 in normal weight patients (95% CI 4.7–4.9), by 4.5 in overweight patients (4.5–4.6) and by 4.3 in obese patients (4.2–4.4) (*p* < 0.001) [4.4 (4.3–4.6), 3.8 (3.5–4.1) and 4.6 (3.9–5.3) in obesity classes I, II, and III, respectively (*p* < 0.001)]. The ODI improved by 30 in normal weight patients (30–31), by 29 in overweight patients (28–29) and by 26 in obese patients (25–27) (*p* < 0.001) [29 (28–29), 25 (22–27) and 27 (22–32) in obesity classes I, II, and III, respectively (*p* < 0.01)]. A total of 3.0% of normal-weight patients, 3.9% of overweight patients and 3.9% of obese patients suffered complications (*p* = 0.047) [3.8%, 4.4%, 3.5% in obesity classes I, II, and III, respectively (*p* = 0.90)].

**Conclusions:**

LDH surgery is also generally associated with favourable outcomes and few complications in patients with morbid obesity.

**Supplementary Information:**

The online version contains supplementary material available at 10.1186/s12891-022-05884-8.

## Background

Lumbar disc herniation (LDH) results in significant costs to society, including both direct health care costs and costs due to sick leave, because of its high prevalence in working-age patients [1–3]. While conservative treatment often leads to satisfactory outcomes [4–6], surgery remains an option in severe cases or when conservative treatment is not successful [7]. LDH surgery is a common surgical procedure; in the US, more than 300 000 procedures are performed annually [8]. Randomized controlled trials (RCTs), as well as other high-quality studies, have also reported favourable outcomes after LDH surgery [9–14], but to further improve the outcome, it is essential to identify predictors of inferior surgical outcomes to reduce surgery in these groups. Previously identified risk factors include old age, chronicity of symptoms and/or preoperative inferior mental health, while the importance of body mass index (BMI) has been debated [15]. Obesity, often defined by the World Health Organization (WHO) as body mass index (BMI) ≥ 30 kg/m^2^ [16], is usually regarded as a risk factor for inferior outcomes after arthroplasty surgery [17], neurosurgery (23), cardiac surgery (24) and aesthetic surgery [18–20], at least partly due to obesity causing other risk factors for comorbidity [21] and anaesthetic complications [22]. Some reports also infer that obesity is a risk factor for inferior outcomes and complications in LDH surgery [23–25], while other studies oppose this view [26, 27].

Based on data in the literature, we hypothesized that obesity could be associated with inferior outcomes and more complications after LDH surgery. We posed the following research questions. (i) Do both obese and nonobese patients with lumbar disc herniation surgery achieve an improvement of clinical relevance [defined as exceeding the minimal clinical difference (MCID)]? (ii) Are there different clinical outcomes and risks for complications in obese and nonobese patients?

## Methods

### The Swedish National Quality Registry SweSpine®

SweSpine is a Swedish National Quality Registry that contains prospectively collected data in conjunction with degenerative lumbar spine surgery [28]. The registry includes 95% of the departments that perform spine surgery in Sweden, with a coverage of 80% and a follow-up of 75% [28]. The patients completed a preoperative questionnaire that assessed baseline demographics and symptoms. These data include body height, body weight, smoking status, and patient-reported outcome measures (PROM), including the Numeric Rating Scale (NRS) for leg and back pain and the Oswestry Disability Index (ODI). BMI is calculated as weight divided by height squared (kg/m^2^). Questionnaires, identical to the preoperative questionnaires, were sent to the patients 1, 2, 5 and 10 years after surgery, with an additional question that evaluates their satisfaction with the surgical outcome (graded as a Likert scale using satisfied, uncertain, or dissatisfied). Perioperative data regarding surgical technique as well as complications during the procedure and the postoperative hospital stay are reported by the operating surgeon. The registered complications are separated into death, dural tear, injury of a nerve root, postoperative haematoma, pulmonary embolism, wound infection, cauda equina syndrome, thrombosis, and “other complication”, all answered Yes or No.

### Study participants

**Fig. 1:**
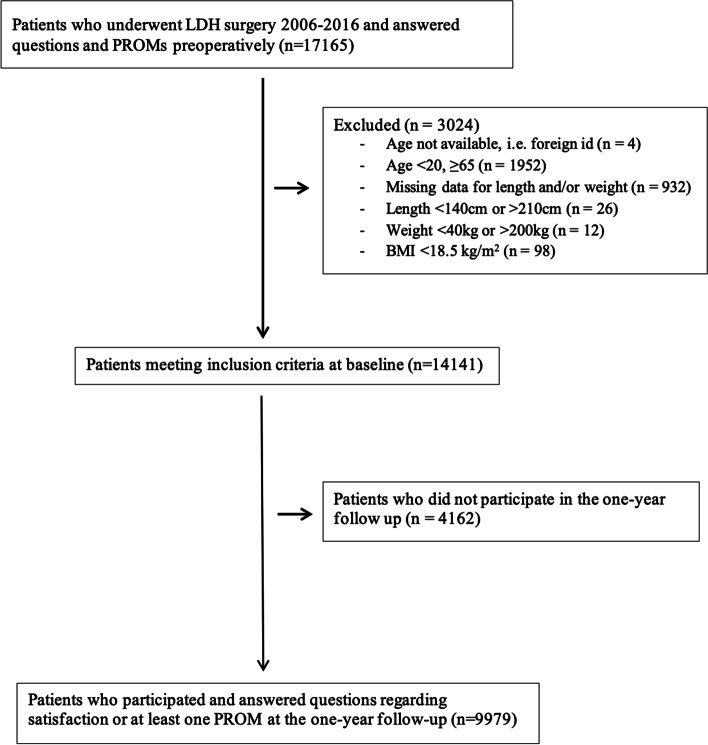
Flow chart of patient inclusion from SweSpine

We identified 17,165 patients in SweSpine who were included due to LDH surgery during the years 2006–2016 with registered preoperative data. The study was designed after the data were already collected, that is, with a retrospective study design. The exclusion criteria were patients with missing age data (*n* = 4), patients age < 20 or ≥ 65 years old (*n* = 1,952), patients with missing body height or body weight data (*n* = 932), and underweight patients (BMI < 18.5 kg/m^2^) (*n* = 98). Due to the large dataset, we chose to exclude 38 patients with nonplausible or very uncommon information on body height or weight. These were individuals with body height < 140 cm or > 210 cm and/or with weight < 40 kg or > 200 kg. These patients had body heights registered between 0.01 and 160 m and weights between 1 and 776 kg, i.e., likely explained by input mistakes. A total of 14,141 patients met our inclusion criteria. At the one-year follow-up, there were 4,162 nonresponders, leaving 9,979 patients with pre- and postoperative data for this report (Fig. 1).

### Drop-out analyses

We performed two drop-out analyses. First, we compared the 9,979 patients (who were included in this report) to the 4,162 patients who only provided preoperative data but not one-year postoperative data (Appendix 1). In the second analysis, we compared the 14,141 patients who provided preoperative data to the 992 patients who provided preoperative data but were excluded due to missing data regarding body weight and/or body height (Appendix 2). We were unable to identify any clinically relevant differences between the groups.

### Outcome variables

Endpoint variables in this report were NRS leg pain, NRS back pain, ODI, and satisfaction one year after surgery, as well as changes from before surgery to one year after in NRS leg and NRS back pain and ODI; all of these were also analysed according to Minimal Clinically Important Differences (MCID). We used this technique because previous studies have shown that the one-year data reflect the final surgical outcome [29]. We further analysed complications in relation to weight class, and we grouped complications into a binary “all complications” variable if a patient had any complication (i.e., patients could have more than one complication).

### Statistical analysis

IBM SPSS version 27 was used for statistical calculations. Descriptive data are presented as numbers, proportions (%) and means ± standard deviations (SD), and inferential data are presented as the means with 95% confidence intervals (95% CI) within brackets. A difference in NRS leg pain equal to or above 3.5, in NRS back pain equal to or above 2.5, and in ODI equal to or above 20 was, as defined by Solberg et al. [30], regarded as MCID. When we analysed the proportion of patients who improved equal to or above MCID, we only included those with preoperative impairment of MCID or more (that is, only those who had a hypothetical chance to improve by MCID or more). We used chi-square and Fischer´s exact tests to test categorical data for uncertainty and analyses of variance (ANOVA) and analyses of covariance (ANCOVA), adjusted for age, gender, smoking and baseline status for the evaluated trait, for continuous data. When using Shapiro‒Wilk´s test to examine departures from normality, we found no values below 0.89. For dichotomous outcomes, we used binary logistic regression. We regarded a *p* value < 0.05 to indicate a statistically significant difference. The study was approved by the Lund regional ethical review board (Ref. no. 2017/158).

## Results

### General outcomes in patients who underwent LDH surgery

Baseline data are presented in Table 1. When evaluating all LDH-operated patients as one group, 78% were satisfied with the surgical outcome one year after surgery, 3.5% of the patients had a complication, and 2.4% a dural tear. The improvement in NRS leg pain was 4.6 ± 3.3, in NRS back pain was 2.2 ± 3.2 and in ODI was 29 ± 21. Among patients with baseline impairment equal to or greater than MCID, 74% showed an improvement equal to or more than MCID in NRS leg pain, 61% in NRS back pain and 69% in ODI.

**Table 1:** Pre- and perioperative data in patients with surgery for lumbar disc herniation (LDH) in relation to body mass index (BMI; g/cm^2^) class. Data are presented as means ± standard deviations or as proportions (%)

	**Normal weight** BMI 18.5–24.9	**Overweight** BMI 25.0–29.9	**Obese class I** BMI 30.0–34.9	**Obese class II** BMI 35.0–39.9	**Obese class III** BMI ≥ 40.0
	*n* = 4156	*n* = 4063	*n* = 1384	*n* = 317	*n* = 59
Age (years)	42 ± 11	44 ± 10	45 ± 10	43 ± 10	41 ± 11
Men/Women	45%/55%	64%/36%	56%/44%	44%/56%	42%/58%
Smokers	16%	14%	16%	19%	19%
Numeric Rating Scale leg pain	6.7 ± 2.4	6.7 ± 2.5	6.7 ± 2.4	7.0 ± 2.5	6.8 ± 2.2
Numeric Rating Scale back pain	4.5 ± 2.9	4.7 ± 2.9	5.0 ± 2.9	5.6 ± 3.0	5.4 ± 2.7
Oswestry Disability Index	47 ± 19	47 ± 18	50 ± 17	53 ± 19	51 ± 17
Acute Surgery	10%	10%	10%	13%	9%
Level of operation					
- L3-L4	3%	5%	6%	7%	2%
- L4-L5	38%	45%	47%	49%	55%
- L5-S1	57%	49%	44%	42%	41%
- Other	1%	1%	3%	2%	2%
Type of surgery					
- Microdiscectomy (open)	47%	44%	40%	38%	36%
- Microdiscectomy (microscope)	43%	44%	45%	45%	44%
- Decompression (open)	3%	4%	5%	7%	12%
- Decompression (microscope)	2%	2%	2%	4%	0%
- Other & not specified	5%	6%	6%	6%	8%

**Fig. 2:**
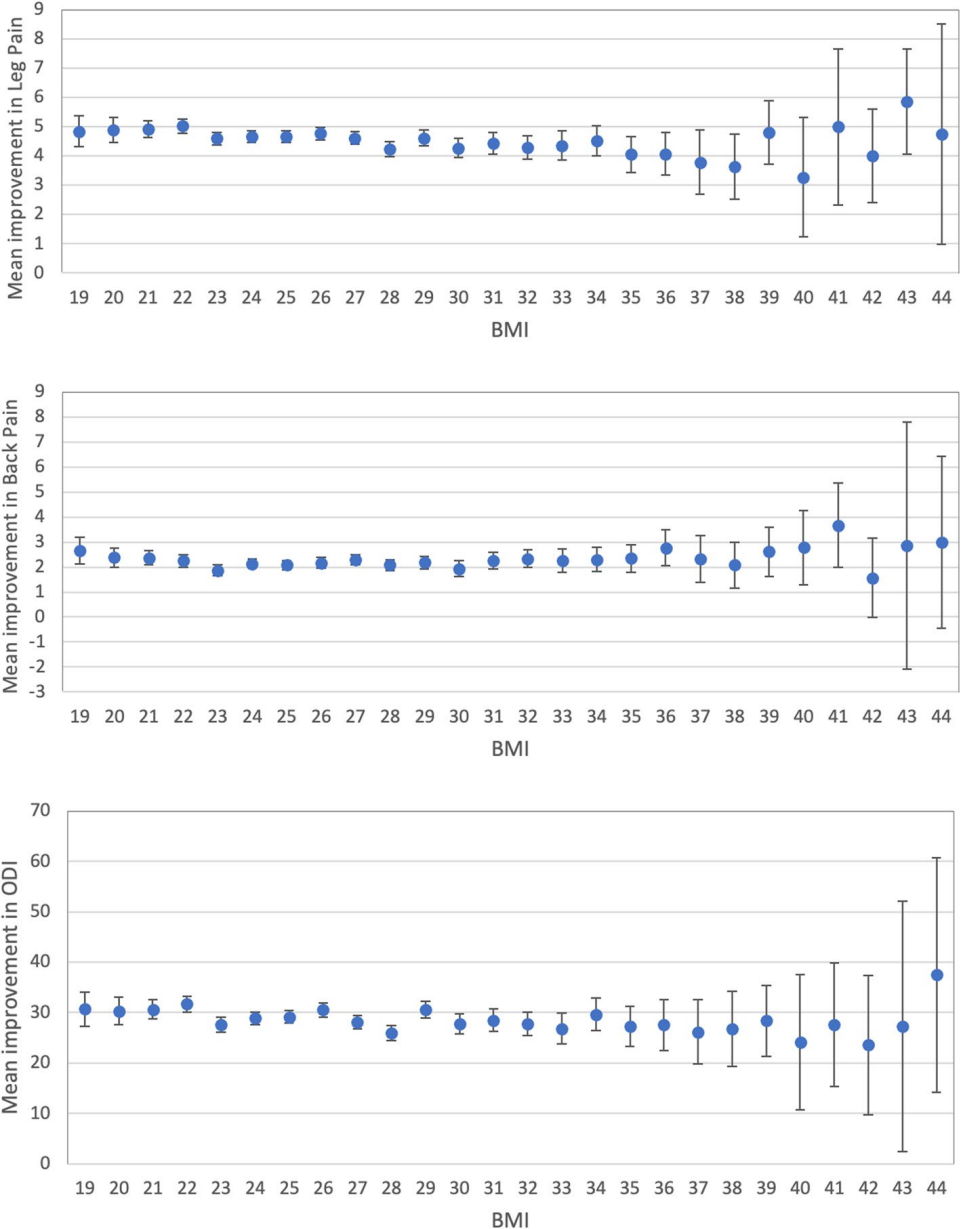
Improvement in Numeric Rating Scale (NRS) leg pain, NRS back pain, and Oswestry Disability Index (ODI) in relation to body mass index (BMI; kg/m^2^) class. BMI mean 19 includes patients with BMI 18.5 – < 19.5, BMI mean 20 those with BMI 19.5—< 20.5 and so on. *n* = 9678 for leg pain, 9676 for back pain and 9681 for ODI. Data are presented as the means with 95% confidence intervals

Associations were found in a linear regression model between preoperative BMI and changes in NRS leg pain [-0.04 (95% CI -0.06, -0.03; *p* < 0.001)] and changes in ODI [-0.18 (95% CI -0.29, -0.08; *p* < 0.001)], but not for changes in NRS back pain [0.01 (95% CI -0.01, 0.02; *p* = 0.34] (Fig. 2).

### Outcomes in normal weight, overweight and obese patients

One year after surgery, 80% of normal weight patients, 77% of overweight patients and 74% of obese patients were satisfied with the surgical outcome (*p* < 0.001). Pre- and postoperative data and improvements in NRS leg pain, NRS back pain and ODI in normal weight, overweight and obese patients are presented in Table 2.

**Table 2:** Pre- and one-year postoperative data and improvement from before to one year after surgery in Numeric Rating Scale (NRS) leg pain, NRS back pain, and Oswestry Disability Index (ODI) in patients with lumbar disc herniation (LDH) surgery in relation to body mass index (BMI; g/cm^2^) class. Number of included patients for each variable are presented as n_1 to_ n_3_. Data are presented as means ± standard deviations and as mean differences, adjusted for age, smoking habits, sex, and baseline value for the evaluated variable, with 95% confidence intervals within brackets. Statistically significant differences are bolded

	**Normal weight** BMI 18.5–24.9			**Overweight** BMI 25.0–29.9			**Obese** BMI ≥ 30.0			**Group comparison improvement**
	n_1_ = 4032			n_1_ = 3896			n_1_ = 1690			
	n_2_ = 4029			n_2_ = 3894			n_2_ = 1692			
	n_3_ = 4032			n_3_ = 3900			n_3_ = 1694			
	Pre-operative	Post-operative	Improvement*	Pre-operative	Post-operative	Improvement*	Pre-operative	Post-operative	Improvement*	*P*-value
NRS leg pain (*n* = 9618)^1^	6.7 ± 2.4	1.9 ± 2.5	**4.8 (4.7, 4.9)**	6.7 ± 2.5	2.1 ± 2.7	**4.5 (4.5, 4.6)**	6.8 ± 2.4	2.5 ± 2.9	**4.3 (4.2, 4.4)**	** < 0.001**
NRS back pain (*n* = 9615)^2^	4.5 ± 2.9	2.3 ± 2.6	**2.3 (2.3, 2.4)**	4.7 ± 2.9	2.5 ± 2.6	**2.1 (2.0, 2.2)**	5.1 ± 2.9	2.9 ± 2.8	**1.9 (1.8, 2.1)**	** < 0.001**
ODI (*n* = 9617)^3^	47 ± 19	18 ± 17	**30 (30, 31)**	47 ± 18	19 ± 17	**29 (28, 29)**	51 ± 18	23 ± 19	**26 (25, 27)**	** < 0.001**

*adjusted for age, smoking habits, sex, and baseline value for the evaluated variable

Among patients with baseline impairment of at least MCID, the proportions of patients who improved equal to or greater than MCID were different in normal weight, overweight and obese patients regarding NRS leg pain (*p* < 0.001) and ODI (*p* < 0.01) but not in NRS back pain (*p* = 0.27) (Table 3). In analyses where we adjusted for age, sex, smoking, and baseline status, we found that overweight and obese patients had lower odds for improvement ≥ MCID in NRS leg pain and ODI than normal weight patients, while the odds for improvement in NRS back pain were similar (Table 3).

**Table 3:** Among patients with a preoperative impairment ≥ Minimal Clinical Important Difference (MCID), proportion of patients who improved by MCID or more after lumbar disc herniation (LDH) surgery in relation to body mass index (BMI; g/cm^2^) class. Numbers of included patients for each variable are presented as n_1 to_ n_3_. Data are presented as proportions (%) and Odds Ratio (OR), adjusted for age, smoking habits, and sex, with 95% confidence intervals within brackets. All ORs are calculated using the proportion that reached improvement ≥ MCID among normal weighted patients as reference cohort. Statistically significant differences are bolded

	**Normal weight** BMI (18.5–24.9)	**Overweight** BMI (25.0–29.9)		**Obese** BMI (≥ 30.0)		**Group comparison proportions improved ≥ MCID**
	n_1_ = 3548	n_1_ = 3409		n_1_ = 1503		
	n_2_ = 2721	n_2_ = 2760		n_2_ = 1292		
	n_3_ = 3826	n_3_ = 3731		n_3_ = 1645		
	Proportions improved ≥ MCID	Proportions improved ≥ MCID	OR for gaining improvement ≥ MCID compared to the reference cohort	Proportions improved ≥ MCID	OR for gaining improvement ≥ MCID compared to the reference cohort	*P*-value
NRS leg pain (*n* = 8460)^1^	77%	73%	**0.8 (0.7, 0.90)**	69%	**0.7 (0.6, 0.8)**	** < 0.001**
NRS back pain (*n* = 6773)^2^	62%	61%	0.9 (0.8, 1.04)	59%	0.8 (0.9, 1.01)	0.27
ODI (*n* = 9202)^3^	71%	69%	**0.9 (0.8, 0.97)**	66%	**0.8 (0.7, 0.91)**	** < 0.01**

A total of 3.0% of normal-weight patients, 3.9% of overweight patients, and 3.9% of obese patients had some complications (*p* = 0.047) (Table 4). The corresponding proportions of patients with dural tears were 1.9%, 2.6%, and 3.1%, respectively (*p* = 0.02) (Table 4).

**Table 4:** Proportion of patients with complications after lumbar disc herniation (LDH) surgery in relation to body mass index (BMI; g/cm^2^) class. Number of included patients for each variable are presented as n_1 to_ n_3_. Data are presented as proportions (%) and Odds Ratio [(Exp(B)], with 95% confidence intervals in brackets. All ORs are calculated using the proportion that reached improvement ≥ MCID among normal weighted patients as reference cohort. Statistically significant differences are bolded

	**Normal weight** BMI 18.5–24.9	**Overweight** BMI 25.0–29.9	**Obese** BMI (≥ 30.0)	***P*-value**
	n_1_ = 4146	n_1_ = 4052	n_1_ = 1745	
	n_2_ = 4145	n_2_ = 4050	n_2_ = 1745	
	n_3_ = 4156	n_3_ = 4063	n_3_ = 1760	
Complications registered by surgeon				
- Dural tear (*n* = 9945)^1^	1.9%	2.6%	3.1%	**0.02**
- Injury of nerve root (*n* = 9940)^2^	0.2%	0.1%	0.3%	0.15
- Other & not specified (*n* = 9979)^3^	0.9%	1.2%	0.7%	0.20
- All complications (*n* = 9979)^3^	3.0%	3.9%	3.9%	**0.047**
Odds Ratio		OR compared to the reference cohort	OR compared to the reference cohort	
- All complication (*n* = 9979)^3^	*–––*	**1.3 (1.04, 1.7)**	1.3 (0.99, 1.8)	–-
- Dural tear (*n* = 9945)^1^	*–––*	**1.4 (1.02, 1.8)**	**1.6 (1.1, 2.3)**	**–-**

### Outcomes in relation to obesity class

One year after surgery, 75% of patients with class I obesity, 71% of patients with class II obesity and 75% of patients with class III obesity were satisfied with the surgical outcome (*p* = 0.43). Pre- and postoperative data and improvements in NRS leg pain, NRS back pain and ODI in relation to obesity class are presented in Table 5.

**Table 5:** Pre- and one-year postoperative data and improvement from before to one year after surgery in Numeric Rating Scale (NRS) leg pain, NRS back pain, and Oswestry Disability Index (ODI) in obese patients with lumbar disc herniation (LDH) surgery in relation to body mass index (BMI; g/cm^2^) class. Number of patients included for each variable are presented as n_1 to_ n_3_. Data are presented as means ± standard deviations and as mean differences, adjusted for age, smoking habits, sex, and baseline value for the evaluated variable, with 95% confidence intervals within brackets. Statistically significant differences are bolded

	**Obese class I** BMI 30.0–34.9			**Obese class II** BMI 35.0–39.9			**Obese class III** BMI ≥ 40.0			**Group comparison improvement**
	n_1_ = 1329			n_1_ = 304			n_1_ = 57			
	n_2_ = 1328			n_2_ = 307			n_2_ = 57			
	n_3_ = 1332			n_3_ = 305			n_3_ = 57			
	Pre-operative	Post-operative	Improvement*	Pre-operative	Post-operative	Improvement*	Pre-operative	Post-operative	Improvement*	*p*-value
NRS leg pain (*n* = 1690)^1^	6.7 ± 2.4	2.3 ± 2.8	**4.4 (4.3, 4.6)**	7.0 ± 2.5	3.1 ± 3.2	**3.8 (3.5, 4.1)**	6.8 ± 2.2	2.1 ± 2.7	**4.6 (3.9, 5.3)**	** < 0.001**
NRS back pain (*n* = 1692)^2^	5.0 ± 2.9	2.8 ± 2.8	**2.3 (2.1, 2.4)**	5.6 ± 3.0	3.2 ± 2.9	**2.2 (1.9, 2.5)**	5.4 ± 2.7	2.8 ± 2.8	**2.6 (1.9, 3.3)**	0.50
ODI (*n* = 1694)^3^	50 ± 17	22 ± 19	**29 (28, 29)**	53 ± 19	27 ± 20	**25 (22, 27)**	51 ± 17	25 ± 21	**27 (22, 32)**	** < 0.01**

*adjusted for age, smoking habits, sex, and baseline value for the evaluated variable

Among patients with baseline impairment of at least the MCID, the proportions of patients who had an improvement equal to or greater than the MCID were different in patients with class I, II and III obesity with respect to NRS leg pain (*p* < 0.01) but not NRS back pain (*p* = 0.43) or the ODI (*p* = 0.15) (Table 6). In the analyses that were adjusted for age, sex, smoking and baseline status, we found that patients with class II obesity had lower odds for improvement ≥ MCID in NRS leg pain but not lower odds for improvement in NRS back pain or ODI than patients with class I obesity (Table 6). Patients with class III obesity had similar odds for improvement in NRS leg pain, NRS back pain and ODI as patients with class I obesity (Table 6).

**Table 6:** Among obese patients with preoperative impairment ≥ Minimal Clinical Important Difference (MCID), proportion of patients who improved by MCID or more after lumbar disc herniation (LDH) surgery in relation to by body mass index (BMI; g/cm^2^) class. Numbers of included patients for each variable are presented as n_1 to_ n_3_. Data are presented as proportions (%) and Odds Ratio (OR), adjusted for age, smoking habits, and sex, with 95% confidence intervals within brackets. All ORs are calculated using the proportion that reached improvement ≥ MCID among obese class I patients as reference cohort. Statistically significant differences are bolded

	**Obese class I** BMI 30.0–34.9	**Obese class II** BMI 35.0–39.9		**Obese class III** BMI ≥ 40.0		**Group comparison proportions improved ≥ MCID**
	n_1_ = 1177	n_1_ = 273		n_1_ = 53		
	n_2_ = 999	n_2_ = 245		n_2_ = 48		
	n_3_ = 1297	n_3_ = 292		n_3_ = 56		
	Proportions improved ≥ MCID	Proportions improved ≥ MCID	OR for gaining improvement ≥ MCID compared to reference cohort	Proportions improved ≥ MCID	OR for gaining improvement ≥ MCID compared to reference cohort	*P*-value
NRS leg pain (*n* = 1503)^1^	70%	60%	**0.6 (0.5, 0.8)**	74%	1.1 (0.6, 2.1)	** < 0.01**
NRS back pain (*n* = 1292)^2^	58%	63%	1.3 (0.9, 1.7)	60%	1.1 (0.6, 2.0)	0.43
ODI (*n* = 1645)^3^	67%	62%	0.8 (0.6, 1.03)	70%	1.1 (0.6, 1.9)	0.15

A total of 3.8% of patients with class I obesity, 4.4% of patients with class II obesity, and 3.5% of patients with class III obesity had at least one complication (*p* = 0.90) (Table 7). The proportions of patients with dural tears were 3.0%, 3.5% and 3.5%, in patients with class I, II, and III obesity, respectively (*p* = 0.72) (Table 7).

**Table 7:** Proportion of obese patients with complications after lumbar disc herniation (LDH) surgery in relation to body mass index (BMI; g/cm^2^) class. Number of patients included for each variable are presented as n_1 to_ n_3_. Data are presented as proportions (%) and Odds Ratio [(Exp(B)] with 95% confidence intervals in brackets. All ORs are calculated using the proportion that reached improvement ≥ MCID among obese class I patients as reference cohort

	**Obese class I** BMI 30.0–34.9	**Obese class II** BMI 35.0–39.9	**Obese class III** BMI ≥ 40.0	***P*-value**
	n_1_ = 1373	n_1_ = 317	n_1_ = 57	
	n_2_ = 1372	n_2_ = 316	n_2_ = 57	
	n_3_ = 1384	n_3_ = 317	n_3_ = 59	
Complications registered by surgeon				
- Dural tear (*n* = 1747)^1^	3.0%	3.5%	3.5%	0.72
- Injury of nerve root (*n* = 1745)^2^	0.4%	0.3%	-	1.00
- Other & not specified (*n* = 1760)^3^	0.7%	1.3%	-	0.54
- All complications (*n* = 1760)^3^	3.8%	4.4%	3.5%	0.90
Odds Ratio		OR compared to the reference cohort	OR compared to the reference cohort	
- All complications (*n* = 1760)^3^	*––-*	1.2 (0.6, 2.1)	0.9 (0.2, 3.7)	–
- Dural tear (*n* = 1747)^1^	*–––*	1.2 (0.6, 2.3)	1.2 (0.3, 5.0)	–

## Discussion

This study indicates that LDH surgery in obese patients is associated with statistically inferior improvement compared with overweight patients and that the outcome in overweight patients is statistically significantly inferior compared to normal weight patients. However, there is no indication that patients with class III obesity had inferior improvement compared to those with class I obesity. It must also be emphasized that the mean improvement in NRS leg pain and ODI was beyond MCID in all BMI groups and that the proportion of patients who recovered equal to or above the MCID level was high in all BMI groups. Furthermore, the study shows that the proportion of patients with complications was low in all BMI groups. In summary, the clinical relevance of the differences in this study should be regarded as minor. When discussing the possible influence of BMI, we must also emphasize that many other factors are important for the final outcome, as are the types of surgery performed and the competence of the surgeons.

The predominantly favourable outcome of LDH surgery in obese patients is supported in the literature. The Spine Patient Outcomes Research Trial (SPORT) showed that the outcomes in obese patients were inferior compared to the outcomes in normal weight patients after both nonoperative and surgical treatment. Additionally, the relative differences in outcomes after surgery and nonoperative treatment were not affected by BMI, and there were no group differences in self-rated improvement or patient satisfaction [25]. The study included 306 obese patients who were stratified into BMI classes below and above 30 kg/m^2^, something that made it impossible to evaluate the outcome in patients with different degrees of obesity. Furthermore, Madsbu et al. reported in a study that included 914 obese patients who, similar to nonobese patients, obese patients improved, even if they had more complications [26]. Our study now adds to our knowledge when reporting that patients in class III obesity patients in general also have favourable outcomes after LDH surgery.

The strengths of our study include the large sample that prospectively evaluated improvement data and end result of LDH surgery in patients with different degrees of obesity. In fact, this is the first publication that specifically evaluates the outcome by PROMs in a larger cohort of morbidly obese patients. Furthermore, inclusion of prospectively collected nationwide PROM data makes it possible to discuss the outcome in an unselected population of LDH-operated patients, thereby presenting the outcome in the general health care setting rather than in highly specialized units or in narrowly defined patient cohorts.

Limitations of this study include the inability to draw conclusions regarding causality, whether more or fewer patients with obesity should undergo an operation, or when the surgeon should be careful when operating on a patient with obesity. The study design also carries the risk of selection bias. Although the drop out analysis revealed no major relevant group differences and we adjusted for baseline impairment in group comparisons, there may still be selection bias in other factors of importance that we did not compare, as well as other confounders that we did not adjust for. However, a previous study found that the drop-out frequency from SweSpine, with a magnitude similar to the dropout magnitude in our study, did not affect the conclusions [31]. Another concern is the possible underreporting of complications [32]. However, we find it unlikely that this would be different in the different BMI classes. Even if our study is the largest sample of prospectively evaluated LDH-operated patients with class III obesity, an even larger sample size would have been advantageous to reduce the risk of type II errors. Another weakness is that we cannot explain the inferior outcome in patients with class II obesity. However, we speculate that the preoperative selection criteria for surgery could have been different in patients with obesity classes II and III or that the difference occurred by chance. Other weaknesses include the inability to compare the outcome by different surgical techniques and that the possible use of different surgical techniques in different populations (normal vs. obese) may influence our conclusions. We can further not access surgeons' rationale, X-rays and several other preoperative factors that may alter the surgeon's decision regarding preoperative decisions and surgical techniques.

We conclude that not only normal weight patients but also overweight patients and those with class I, II, and III obesity in general have favourable outcomes after LDH surgery. On a group level, we found statistically significantly poorer outcomes and more complications in overweight and obese patients than in normal-weight patients, but the differences were of minor clinical significance, as all groups improved on average by more than MCID.

## Supplementary Information

**Additional file 1: Appendix 1.** Preoperative data in patients who responded to both the preoperative and the one-year postoperative questionnaire (responders) and in those who answered the preoperative but not the one-year postoperative questionnaire (non-responders). Data are shown as means ± SD or proportions (%).

**Additional file 2: Appendix 2.** Preoperative data in patients with weight and length registered and in patients with missing data for length and/or weight. Data are presented as mean ± SD or proportions (%).

## Data Availability

Data used in this study can be obtained upon request and approval from the Swedish National Spine registry (Swespine).

## Abbreviations

LDH: Lumbar disc herniation
ODI: Oswestry Disability Index
NRS: Numeric Rating Scale
MCID: Minimal Clinical Important Difference
BMI: Body Mass Index. calculated as weight divided by height squared (kg/m^2^)
PROM: Patient Reported Outcome Measures
Swespine: Swedish National Spine Registry
ANOVA: Analysis Of Variance
ANCOVA: Analysis Of Covariance
