# Supplementary material for: A study comparing outcomes between obese and nonobese patients with lumbar disc herniation undergoing surgery: a study of the Swedish National Quality Registry of 9979 patients
Source: BMC Musculoskelet Disord. 2022 Oct 22;23:931. doi: 10.1186/s12891-022-05884-8 (PMC9587539; doi:10.1186/s12891-022-05884-8)
Supplement: Supplementary file 1 — Additional file 1: Appendix 1. Preoperative data in patients who responded to both the preoperative and the one-year postoperative questionnaire (responders) and in those who answered the preoperative but not the one-year postoperative questionnaire (non-responders). Data are shown as means ± SD or proportions (%). [file 12891_2022_5884_MOESM1_ESM.docx]

**Appendix 1**: Preoperative data in patients who responded to both the preoperative and the one-year postoperative questionnaire (responders) and in those who answered the preoperative but not the one-year postoperative questionnaire (non-responders). Data are shown as means ± SD or proportions (%).

|  | **Responders** | **Non-Responders** |
| --- | --- | --- |
|  | n = 9979 | n = 4162 |
| Age (years) | 43.6 ± 10.6 | 40.9 ± 10.1 |
| Body Mass Index (kg/m^2^) | 26.4 ± 4.2 | 26.8 ± 4.5 |
| Men/Women (%) | 54/46 | 61/39 |
| Smokers (%) | 15 | 22 |
| Numeric Rating Scale (NRS) leg pain | 6.7 ± 2.4 | 6.8 ± 2.4 |
| Numeric Rating Scale (NRS) back pain | 4.7 ± 2.9 | 5.1 ± 2.9 |
| Oswestry Disability Index (ODI) | 48 ± 18 | 49 ± 18 |
