# Supplementary material for: A study comparing outcomes between obese and nonobese patients with lumbar disc herniation undergoing surgery: a study of the Swedish National Quality Registry of 9979 patients
Source: BMC Musculoskelet Disord. 2022 Oct 22;23:931. doi: 10.1186/s12891-022-05884-8 (PMC9587539; doi:10.1186/s12891-022-05884-8)
Supplement: Supplementary file 2 — Additional file 2: Appendix 2. Preoperative data in patients with weight and length registered and in patients with missing data for length and/or weight. Data are presented as mean ± SD or proportions (%). [file 12891_2022_5884_MOESM2_ESM.docx]

**Appendix 2**: Preoperative data in patients with weight and length registered and in patients with missing data for length and/or weight. Data are presented as mean ± SD or proportions (%).

|  | **Patients with length and weight registered** | **Patients with length and/or weight missing** |
| --- | --- | --- |
|  | n = 14141 | n = 932 |
| Age (years) | 42.8 ± 10.5 | 43.2 ± 10.8 |
| Men/Women (%) | 56/44 | 52/48 |
| Smokers (%) | 17 | 23 |
| Numeric Rating Scale (NRS) leg pain | 6.7 ± 2.4 | 6.8 ± 2.4 |
| Numeric Rating Scale (NRS) back pain | 4.8 ± 2.9 | 5.0± 2.9 |
| Oswestry Disability Index (ODI) | 48 ± 18 | 49 ± 17 |
